# ABCE1: A special factor that orchestrates translation at the crossroad between recycling and initiation

**DOI:** 10.1080/15476286.2016.1269993

**Published:** 2017-05-12

**Authors:** Eder Mancera-Martínez, Jailson Brito Querido, Leos Shivaya Valasek, Angelita Simonetti, Yaser Hashem

**Affiliations:** a CNRS, Architecture et Réactivité de l'ARN UPR9002, Université de Strasbourg, Strasbourg, France; b Laboratory of Regulation of Gene Expression, Institute of Microbiology ASCR, Prague, Czech Republic

**Keywords:** ABCE1, cryo-EM, translation, recycling, ribosome

## Abstract

For many years initiation and termination of mRNA translation have been studied separately. However, a direct link between these 2 isolated stages has been suggested by the fact that some initiation factors also control termination and can even promote ribosome recycling; i.e. the last stage where post-terminating 80S ribosomes are split to start a new round of initiation. Notably, it is now firmly established that, among other factors, ribosomal recycling critically requires the NTPase ABCE1. However, several earlier reports have proposed that ABCE1 also somehow participates in the initiation complex assembly. Based on an extended analysis of our recently published late-stage 48S initiation complex from rabbit, here we provide new mechanistic insights into this putative role of ABCE1 in initiation. This point of view represents the first structural evidence in which the regulatory role of the recycling factor ABCE1 in initiation is discussed and establishes a corner stone for elucidating the interplay between ABCE1 and several initiation factors during the transit from ribosomal recycling to formation of the elongation competent 80S initiation complex.

## Introduction

### Eukaryotic translation initiation

Eukaryotic initiation starts with the assembly of a ternary complex (TC) consisting of Met-tRNA_i_, eIF2 and GTP, which is recruited to the 40S subunit with help of eIFs 1, 1A and the 12-subunit eIF3 (a-m subunits excluding eIF3j^1^) to form a 43S preinitiation complex (43S PIC).^2-5^ The binding of these factors to the 40S subunit produces an “open” state to which the TC binds readily.^6^ The eIF2-GTPase-activating protein eIF5 binds the 43S PIC along with the TC but its location within the 43S complex is still unknown. The 43S PIC is then recruited to the capped 5′-end of an mRNA bound to the cap-binding complex formed by eIF4F, eIF4A and eIF4B. Thus formed 48S initiation complex (48S IC), in the open P^OUT^ conformation in which Met-tRNA_i_ is not fully accommodated in the P-site, scans the 5′-region until it encounters the AUG start codon in a suitable initiation context. The mRNA attachment to the 43S PIC triggers the relocation of eIF3b most likely with its closely associated eIF3i and g subunits from the solvent side to the intersubunit face of the 40S subunit^7^ to bind below the TC in the proximity of helix 44 (h44), which may activate the stimulatory roles of this eIF3b-g-i trimeric eIF3 module in scanning.^8,9^ This relocation was also observed in recent cryo-electron microscopy (cryo-EM) reconstructions of a partial yeast 48S IC (py48S IC),^10^ but was at first attributed to subunits i and g of eIF3^10^ before being reassigned to eIF3b in our recent late-stage 48S IC relative work^7^ (please see our discussion therein). The AUG recognition triggers the scanning arrest of the 48S complex inducing a conformational change of the 40S head relative to its body back to the “closed” state in which Met-tRNA_i_ is fully engaged in the P-site (the so called P^IN^ state).^10,11^ According to the model proposed by others (reviewed in Hinnebusch 2014^5^) and built upon in our latest work,^7^ eIF1 is, as a result of start-codon recognition, released or at least relocated from the P-site along with the C-terminal tail of eIF1A (eIF1A-CTT),^12-15^ and most probably also along with the eIF3b-g-i module that is no longer needed to promote scanning.^7^ In any case, the eIF1 move is believed to allow an irreversible release of free Pi from GTP hydrolyzed on eIF2 upon AUG recognition,^16^ resulting in dissociation of eIF2 and eIF5 from the late-stage IC, which is composed of the 40S subunit, mRNA, eIF1A, eIF3, fully accommodated Met-tRNA_i_ in the P-site, and possibly also eIF4F (reviewed in Valásek, 2012).^17^ The dissociation of eIF2 exposes one of the binding sites for eIF5B (which binds the CCA end of the Met-tRNA_i_^18,19^) and the interplay between eIF5B and Met-tRNA_i_ allows each other to acquire their 60S-binding-competent conformations.^18-20^ Finally, the interaction of eIF5B with the ribosome promotes 60S joining and after GTP hydrolysis on eIF5B, this factor together with eIF1A leaves the 80S IC rendering the ribosome elongation competent.^21,22^

In our latest work on the late-stage 48S IC,^7^ we mistakenly assigned eIF3g and i subunits to densities observed close to the GTPase binding site of the 40S and proposed that they may prevent a premature 60S subunit joining. Here we propose a re-interpretation of this cryo-EM reconstruction by assigning these densities to ABCE1 instead. We thus link for the first time recycling and initiation stages in a single structure and discuss our new interpretation and its implications in the regulation of translation initiation in eukaryotes. In detail, we attempt to address some of the long-standing questions on how ABCE1 interacts with the constituents of the PIC at different stages of the process and how these interaction networks govern the progress of the entire initiation pathway. Finally, we also discuss how ABCE1 undergoes its NTP hydrolytic cycles to regulate initiation according to the energy level in the cell.

### ABCE1 is able to bind to translation initiation complexes in vivo

**Figure 1.**
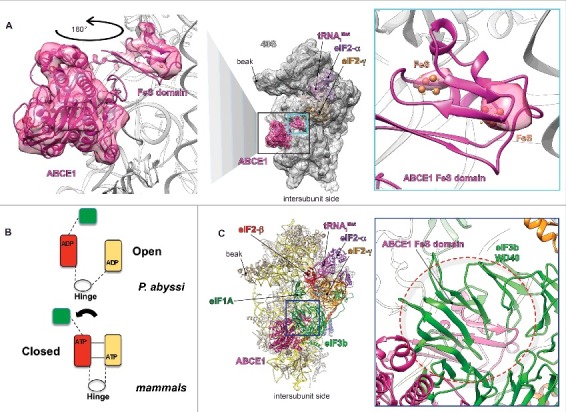
Fitting of ABCE1 in our late-stage m48S initiation complex.^7^ (A) Atomic model of the m48S late-stage IC fitted in its cryo-EM segmented map (middle panel), focused on the densities attributed to ABCE1, seen from the intersubunit face. Right panel, close-up on the atomic model ABCE1 fitted in its density map. Left panel, Blow-up on the FeS clusters domain of ABCE1 at high contour-threshold of the map showing the coincidence of the latter clusters with the highest density spots, which supports our model. (B) Schematic model of the observed conformational rearrangement of ABCE1 in the m48S late-stage complex compared with the P. abyssi crystal structure. (C) Partial overlapping between eIF3b subunit and the FeS domain of ABCE1, seen from the intersubunit face, highlighted by dashed cicle.

We recently published a cryo-EM reconstruction at 5.86Å of the late-stage m48S IC,^7^ purified from rabbit reticulocytes treated with non-hydrolysable GTP analog, which blocks translation at the AUG decoding stage by preventing GTP hydrolysis on eIF2. The complex showed the presence of densities at the intersubunit face of the 40S at the universally conserved GTPase binding site of the 40S ribosomal subunit^7^ that we initially attributed to subunits i and g of the eIF3 complex. The presence of these 2 small subunits of eIF3 was interpreted as the result of their putative relocation to the intersubunit face at the late initiation stage to act as the ribosome anti-association force preventing the premature end of the initiation process. The GTPase binding region is the site where numerous well-characterized translation-related GTPases bind to operate as the key regulators during initiation, elongation, termination and ribosome recycling. However, taking into account the resolution limits of our Cryo-EM reconstructions, where side-chains and single bases are unresolved, several alternative interpretations of our m48S IC structure were possible. Indeed, further analyses of this structure revealed that the ABCE1 crystal structure from *Pyrococcus abyssi*^23^ can, after several extreme but natural conformational changes outlined below, fit into the m48S IC intersubunit densities with much greater consistency than eIF3i and eIF3g subunits. In fact, it perfectly matches with the observed secondary structure elements of ABCE1 (Fig. 1a). Interestingly, we deduce that the FeS cluster domain of ABCE1 adopts a radically different conformation in our structure than that observed in the context of the recycling 80S complex^24^; i.e., it is rotated toward h44 where it is stably anchored. To validate this new interpretation we demonstrate here that the 2 FeS clusters of ABCE1 are in perfect fit with 2 high-density spots that are clearly distinguishable in our map (Fig. 1a), in agreement with their expected opacity to electrons. Moreover, the proteomic profile of the GMP-PNP stalled m48S IC displayed a large amount of ABCE1, which we initially overlooked, since ABCE1 was not considered to associate with the 48S PIC at such a late initiation stage. Although both interpretations – eIF3i+g or ABCE1 – are at the resolution of our structure in theory possible, a deeper analysis of this structure and thorough search of the relevant literature, as well as our discussions over unpublished, made us to confidently reassign these densities to ABCE1.

Cryo-EM reconstructions of termination- or recycling-related complexes revealed that the NBD-containing core of ABCE1, when bound to ATP, occupies the GTPase-binding center and its FeS cluster domain binds and stabilizes the release factor eRF1^25^ or the archaeal mRNA surveillance factor aPelota,^14^ respectively. Strikingly, compared with the *Pyrococcus abyssi*^23^ crystal structure, the new tentative model of the m48S IC proposed here indicates that ABCE1 could adopt a novel conformation, unobserved previously, where the NDB binding domains are in a fully closed conformation and the FeS cluster domain is rotated by ∼180° to acquire a proper orientation for direct binding to the 40S h44 (Fig. 1b). In accord, a recent molecular study reported that the FeS cluster domain of archaeal AMP-PNP-stalled ABCE1 is essential for its interaction with the small subunit.^26^ Hence, our re-interpretation indicates that NTP-bound ABCE1 can adopt a completely closed conformation and highly likely binds to the eukaryotic 48S IC after start codon recognition (as demonstrated by the conformation of the 40S head and the solid density for the codon-anticodon complex^5^), in agreement with earlier reports suggesting that this recycling factor may also participate in initiation.^27-29^ This re-interpretation thus reinforces these reports by proposing that ABCE1 acts as a subunit anti-association factor, and, as such, could be considered as a novel *bona fide* translation initiation factor. In the following chapters we will discuss possible implications of the ABCE1 involvement in ribosomal recycling and in translation initiation; i.e., in the role that has remained underexplored so far, as well as during the transition between these 2.

### The role of ABCE1 as a recycling factor at the interface between recycling and initiation

The mRNA translation process is thought to be cyclic and thus the by-default pathway for a new round of protein synthesis after termination is the reuse of post-termination ribosomes.^30^ After termination, the post-termination ribosomal complexes (post-TC) comprising mRNA and deacylated tRNA-bound ribosomes are split into free 60S subunits and the deacylated tRNA and mRNA-bound 40S subunits in a process called ribosome recycling.^31^ It was shown that the highly conserved and essential ABC-type NTPase ABCE1 functions as the ribosome recycling factor over a wide range of Mg^2+^ concentrations, and its post-TC dissociation efficiency is enhanced by the presence of eIF6 (a well-described anti-association factor)^32^ and/or eIFs 1, 1A and 3^31^, which most likely prevent ribosomal subunit joining by binding to the post-recycled 60S subunits and 40S complexes, respectively. The requirement for such activities in ribosome splitting stresses out that re-association is the intrinsic behavior of free ribosomal subunits in nature. During recycling, ABCE1 splits post-TCs,^31^ however, the subsequent release of tRNA and mRNA from the recycled 40S subunits requires eIF3, eIF1 and eIF1A. Hence, these canonical initiation factors must bind to the 40S subunits already at the end of the translational cycle; i.e., during ribosomal recycling^33^ that precedes formation of the 43S PIC that they promote. In fact, eIF3 has also been implicated in controlling translation termination and programmed stop codon readthrough^34,35^ suggesting that it comes to the picture even earlier, during formation of pre-termination 80S complexes (see below). Taken together, these observations clearly suggest an existence of a mechanistic link between recycling and initiation.

### NTP hydrolysis is the driving force during ABCE1-mediated recycling

ABCE1 is one of the most conserved proteins in evolution that is encoded by all eukaryotic and archaeal genomes,^3637^ and whose catalytic activity lacks nucleotide specificity.^37^ Unlike the conventional features of ABC enzymes, ABCE proteins are single-chain polypeptides harboring 2 nucleotide binding domains (NBDs), an N-terminal domain containing 2 [4Fe-4S] clusters (FeS cluster domain), and no trans-membrane domain, implying that ABCE1 is not a trans-membrane carrier.^23,38^ The available crystal structures of archaeal ABCE1 show that NBDs adopt the distinctive head-to-tail orientation, in which 2 NTP molecules (ABCE1 can bind and hydrolyze any NTP and not only ATP^37^) are fastened between the Walker A and B motifs of one NBD and the C-loop of the other NBD.^39^ This structural configuration is found in all ABC proteins and allows these enzymes to transform chemical energy derived from its NTP hydrolytic activity into structural rearrangements that can be used as kinetic energy.^40^ The NTP-bound “closed” state of ABCE1 is characterized by an approach of its 2 NBDs, whereas in the ADP-bound “open” conformation, both NBDs move further away from each other.^24,41,42^ It was reported that ABCE1 undergoes a cyclic tweezers-like conformational transition that is driven by the binding and hydrolysis of NTP and the release of Pi. Thus ABCE1 switches from an open to a closed NTP-occluded conformation.^24,41,43^ This transition was suggested to trigger an allosteric effect in its FeS cluster domain inducing conformational changes that are transmitted to its close partners. An alternative/complementary hypothesis considers this conformational change as the mechanical driving force that disrupts some intersubunit bridges during recycling,^24,41,43^ thus shifting the equilibrium toward the dissociation of ribosomal subunits.

### ABCE1 bridges recycling with initiation by binding to initiation factors

New mechanistic insights into the transition between termination, recycling and initiation suggest that recycling and initiation factors link these stages of eukaryotic protein synthesis.^31,34,35,44,45^ Indeed, we have recently demonstrated that eIF3 associates with pre-termination complexes where it interferes with the eRF1 decoding of the third/wobble position of the stop codon. This interference allows incorporation of near-cognate tRNAs with a mismatch at the same position to promote programmed stop codon readthrough.^35^ In fact, an unspecified defect in the fidelity of termination was previously reported also for yeast ABCE1 (RLI1).^46^ In case of a canonical termination, eIF3 promotes, together with eIF1 and eIF1A, ribosomal recycling through an energy-free mechanism that only works at low Mg^2+^ concentrations,^44^ thus in principle connecting the ending and the initial steps of mRNA translation. However, it seems that not only initiation factors are able to cross the boundary between recycling and initiation; based on our re-interpretation the recycling factor ABCE1 can do it too. Several independent studies have shown solid data indicating that ABCE1 binds directly eIF3, eIF5 and eIF2 and, when bound to NTP, it can associate with the small ribosome subunit *in vivo*.^27,34,41,47-49^ It was also observed that the AMP-PNP-bound ABCE1 efficiently associates with the 43S complexes,^50^ however the molecular rearrangement induced by NTP-hydrolysis on ABCE1 and its function in translation initiation has remained an open question.

## Insights into the putative roles of ABCE1 in translation initiation

### ABCE1, a bona-fide initiation factor in eukaryotes that may ensure stable binding of other initiation factors

Owing to the great complexity of the eukaryotic initiation process, it is possible that some important regulatory activities performed by known players have been overlooked due to redundancy and/or an unexpected breadth of their functional range of action. This may very well apply to ABCE1 thanks to its reported intrinsic ability to associate with the small ribosomal subunit in presence of NTP *in vitro* and *in vivo*,^26-28,47,48,57,58^ as well as with initiation factors such as eIF3 (via a and j subunits), eIF5 and eIF2.^27,34,41,47-49^ Hence, these observations could suggest a role of ABCE1 as an NTP-dependent scaffold factor that may ensure stable binding and proper positioning of some initiation factors during translation initiation, perhaps according to its NTP-binding status. In fact, such a stimulatory role for yeast ABCE1/RLI1 in assembly of 43S PICs preinitiation complexes *in vivo* has already been suggested by others.^27^

It is noteworthy that, after particle sorting, one of the cryo-EM reconstructions of the late-stage m48S IC^7^ displayed eIF2-TC, ABCE1, and the eIF3 octamer – lacking densities for eIF3b-g-i module – at its known solvent side location on the 40S with a slightly altered conformation. This observation further supports the idea that ABCE1 can coexist with these eIFs on the late-stage IC after start-codon recognition.^27,28,47^ However, what is the real role of ABCE1 in formation of the 43S PICs, if any, must be thoroughly explored further.

### ABCE1, an anti-association factor of ribosomal subunits

**Figure 2.**
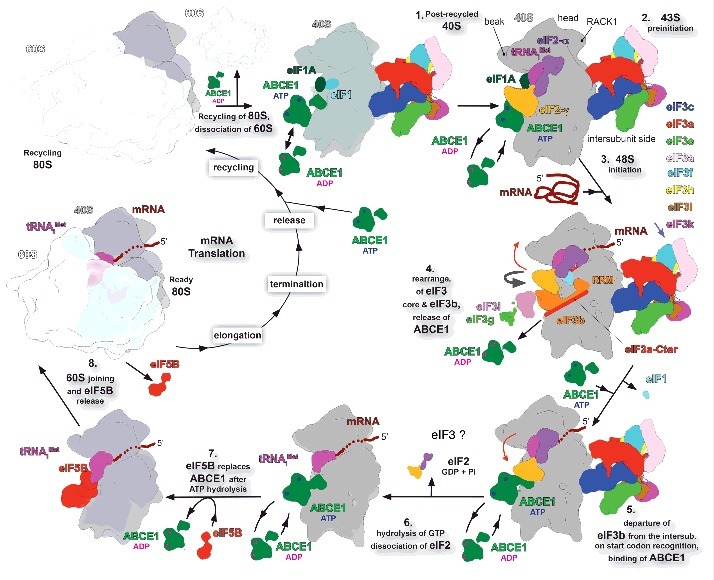
Model of the ABCE1 activity during translation initiation. We propose that the ABCE1, depicted in green, binds to either the 40S–deacylated tRNA–mRNA recycling intermediate along with eIFs 1, 1A and 3, which will then capture the ribosomal recycling stage by producing the free post-recycled 40S complex, or past this step directly to the post-recycled 40S-eIF1-eIF1A-eIF3 complex. During the pre-initiation and initiation stages ABCE1 may act as the critical anti-association factor by cycling on and off the ribosome in an NTP-dependent manner, unless its binding site at the GTPase binding region of the 40S subunit is occupied by other factors such as the eIF3b subunit. At a late initiation stage, eIF5B prevents ABCE1 from further cycling to promote proper subunit joining followed by GTP hydrolysis and its own release. Thick curved gray arrows indicate the eIF3b-g-i module relocation from the solvent to the intersubunit side of the 40S subunit. Red arrow indicates the conformational change of the eIF2-TC upon eIF3b relocation and departure to and from the intersubunit face of the 40S, which may reflect its described previously P^IN^ and P^OUT^ conformations. A question mark indicates an unknown fate of eIF3 that has been proposed to remain bound to the 40S subunit even past subunit joining PMID:18765792; PMID:18056426).

Although NTP hydrolysis of ABCE1 promotes 70S dissociation in archaea, it has been demonstrated that it is not essential for ribosome splitting *per se* but for its own release from the small subunit.^26^ This observation could indicate that a permanent binding of ABCE1 to the 40S subunit during initiation may “freeze” the process at any step. That is why we propose a model where the NTP-bound ABCE1 undergoes several on-off catalytic cycles throughout the initiation process by binding to the GTPase binding site and, upon NTP hydrolysis, dissociating from it. At this point, it is pertinent to reiterate the NTP hydrolysis cycle of ABCE1. As mentioned above, once NTP is loaded onto ABCE1, it adopts a fully closed arrangement, the FeS binding domain rotates and can associate with the small ribosomal subunit.^24,26^ However, upon NTP hydrolysis and Pi release, ABCE1 dissociates from the 40S,^50^ most likely due to drastic conformational transitions where the NDBs distance themselves from each other and the FeS cluster domain swivels away from 40S. This may appear contradictory with its proposed function as an anti-association factor. However, this apparent contradiction disappears, if we assume that ABCE1 is continuously alternating on and off the 40S complexes following the NTP hydrolysis rounds (Fig. 2). In this view, NTP-bound ABCE1 can be recruited to the 40S complexes at any stage of the initiation pathway, unless its binding site is masked, for example by eIF3b (see below), dissuading the 60S subunits from joining till the NTP is hydrolyzed to NDP. The release of NDP-bound ABCE1 is followed by the arrival of another NTP-bound molecule of ABCE1 to keep the cycle running. During the short-lived absences of ABCE1 from the ribosome, critical steps of the initiation pathway could proceed forward as speculated below. Hence, this on-off cycle of ABCE1 could serve as an emergency brake allowing irreversible movement forward only when the pathway is ready for taking the next critical step. At the same time it would prevent premature subunit joining and thus the initiation decay by the tight binding of ABCE1.

According to our model, the initiation cycle starts right after the ribosomal recycling, a stage where the 40S subunit is dissociated from the 60S subunit, de-acetylated tRNA and mRNA, the release of the latter 2 of which is supposed to be aided by eIFs 1, 1A and 3 (Fig. 2, step 1). Thus liberated post-recycled 40S complexes are then ready for formation of the 43S preinitiation complex. At this stage ABCE1 could cycle on and off the 43S complex preventing premature 60S joining; from this perspective it is very likely that ABCE1 binds the 40S subunit very shortly after subunit splitting, perhaps even before the mRNA and tRNA ejection step together with the eIFs mentioned above. To the best of our knowledge, none of the constituents of the 43S PIC occupies the 40S GTPase binding-site, where ABCE1 binds, hence all these factors can co-exist at this early PIC as proposed before (Fig. 2, step 2). The mRNA attachment marks the start of the 48S initiation complex assembly and detonates the re-location of the eIF3b-g-i module to the intersubunit face of 40S in a way that sets the eIF2-TC in the scanning-competent conformation, as described previously^7^ (Fig. 2, steps 3 and 4, respectively). At this stage, the FeS cluster domain of ABCE1 would clash with eIF3b after its relocation below the eIF2-TC if ABCE1 remained attached (Fig. 1c and Fig. 2, step 4). Therefore we believe that ABCE1 will be prevented from cycling in and out of the scanning complex for as long as eIF3b remains bound to the interface side.

Once the start codon is recognized, the eIF3b-g-i module swings away from the 40S intersubunit face, as described previously,^7^ and the eIF2-TC adopts the P^IN^ conformation in the closed state of the preinitiation complex (Fig. 2, step 5). The eIF3b departure from the intersubunit face of the 40S clears up the binding site for ABCE1 FeS domain to re-establish its cycling (Fig. 2, step 5 and 6). Meanwhile, eIF5 stimulated GTP hydrolyses on eIF2 produces irreversible Pi release and these factors leave the 48S PIC (Fig. 2, step 6). It is not known at which exact moment eIF3 leaves the late-stage initiation complexes, in fact it has been proposed to remain bound to the 40S subunit even past subunit joining during a few elongation cycles.^51,52^ Importantly, the ABCE1 is the only factor preventing the premature 60S subunit joining at this stage. The ejection of eIF2 allows eIF5B binding to the GTPase binding-site (Fig. 2, step 7), which again stops the ABCE1 cycling. The arrival of eIF5B promotes the association of the 60S subunit marking the end of the initiation stage by producing the elongation-competent 80S ribosome (Fig. 2, step 8).

### ABCE1, a eukaryotic energy-sensing translational throttle

A supporting rationale for the on-off cycling of ABCE1 could be directly related to the global quantity of NTPs found in a cell at a given time. It is conceivable that ABCE1 could be playing a role of an acceleration throttle by modulating the initiation rates in response to the available energy resources. Interestingly, such a role has been recently reported for another ABC member from the F family, termed Energy-dependent Translational Throttle A (EttA).^53,54^ In compliance with our proposal, EttA cycles on and off from the E-site in the context of the elongating 70S ribosome responding to the global level of NTP in a cell. In addition, a similar NTP-sensing mechanism is used by the RNA polymerase operating on rRNA promoters in bacteria,^55^ and by the RNA polymerase II governing transcription of the IMD2 gene, encoding the yeast inosine monophosphate dehydrogenase (IMPDH) catalyzing the first dedicated step of GTP biosynthesis.^56^ Further studies must be conducted to explore this hypothesis that has the merit of linking the initiation rates to the availability of energy sources.

## Concluding remarks

In this point of view we proposed a new hypothesis featuring ABCE1 as a *bona fide* initiation factor with an important anti-association activity throughout the entire initiation phase. In addition to that we speculated that ABCE1 might also somehow ensure stable binding of several initiation factors while the 43S PIC is formed. Finally, we hypothesized a role for ABCE1 in regulating the initiation rates according to the energy metabolism of a cell. It is very tempting to speculate that the energy level in a cell closely coordinates all ABCE1 roles in initiation, termination and ribosomal recycling, which would make this highly conserved protein one of the main general regulators of the translational output in changing environmental conditions. In any case, it is for certain that our findings with ABCE1 presented here and with eIF3 published before^34,35^ strongly support the idea that there is a highly coordinated communication between individual translational phases. We are thus convinced that a strict separation of translation into its different stages has hidden the versatile nature of several multi-tasking factors including the recycling factor ABCE1 and the initiation factor eIF3, and perhaps many more, at the interface between termination/recycling and initiation. It will be exciting to follow further progress of this field especially with respect to the anticipated diversity of its various factors whose unexpected functions in translation, and even beyond translation, await their discoveries.
